# EML4-ALK Variants: Biological and Molecular Properties, and the Implications for Patients

**DOI:** 10.3390/cancers9090118

**Published:** 2017-09-05

**Authors:** Sarah R. Sabir, Sharon Yeoh, George Jackson, Richard Bayliss

**Affiliations:** Astbury Centre for Structural Molecular Biology, School of Molecular and Cellular Biology, Faculty of Biological Sciences, University of Leeds, Leeds LS2 9JT, UK; s.sabir@leeds.ac.uk (S.R.S.); yeoh.sharon@gmail.com (S.Y.); gfjjackson@gmail.com (G.J.)

**Keywords:** anaplastic lymphoma kinase, echinoderm microtubule-associated protein, non-small cell lung cancer, tyrosine kinase inhibitor

## Abstract

Since the discovery of the fusion between EML4 (echinoderm microtubule associated protein-like 4) and ALK (anaplastic lymphoma kinase), EML4-ALK, in lung adenocarcinomas in 2007, and the subsequent identification of at least 15 different variants in lung cancers, there has been a revolution in molecular-targeted therapy that has transformed the outlook for these patients. Our recent focus has been on understanding how and why the expression of particular variants can affect biological and molecular properties of cancer cells, as well as identifying the key signalling pathways triggered, as a result. In the clinical setting, this understanding led to the discovery that the type of variant influences the response of patients to ALK therapy. Here, we discuss what we know so far about the EML4-ALK variants in molecular signalling pathways and what questions remain to be answered. In the longer term, this analysis may uncover ways to specifically treat patients for a better outcome.

## 1. Introduction

Lung cancer remains one of the most common types of cancer, accounting for 13% of all cancers diagnosed worldwide [1]. Due to late stage diagnosis, treatment tends to be more palliative, and so it is also the most common cause of cancer death [2]. Unfortunately, survival rates have changed little over the past 40 years, with only 5% of patients surviving for more than 10 years post-diagnosis [3].

More than 80% of lung cancer cases are categorised as non-small cell lung cancer (NSCLC), and the majority of these cases are adenocarcinoma in histology. Whilst the main proportion of lung cancer cases are caused by smoking, around 25% are seen in patients that have little or no smoking history [4]. Genetic analysis has increased our knowledge of the molecular events that lead up to lung cancer, and this has allowed us to identify key driver mutations involved. As a result, both the treatment and outcome of patients has drastically changed in recent years, and molecular screening is now a routine procedure for NSCLC.

Two of the common clinical screens in NSCLC are for the presence of mutations in EGFR, or the presence of oncogenic gene translocations, such as EML4-ALK, resulting in treatment with an appropriate kinase inhibitor. Treatment is therefore more personalised and this has resulted in an improvement in progression-free survival (PFS) and quality of life when compared to standard cytotoxic chemotherapy [5,6].

Fusion between EML4 (echinoderm microtubule associated protein-like 4), a microtubule-associated protein, and ALK (anaplastic lymphoma kinase), a tyrosine kinase receptor belonging to the insulin receptor superfamily, was the first oncogenic fusion to be detected in lung cancer [7]. Fusion of EML4 to the kinase domain of ALK results in abnormal signalling and consequently increased cell growth, proliferation, and cell survival. Patients expressing this fusion are therefore treated with an ALK inhibitor such as crizotinib, ceritinib, or alectinib. Whilst these treatments have been very effective, patient response is varied and secondary mutations often lead to relapse within a year [8].

We now know that EML4-ALK is expressed as at least 15 different variants and emerging evidence has shown that expression of particular variants directly impacts the response of patients to ALK inhibitors. Current research aims to examine more closely how variants can affect the therapeutic response through understanding differences in their biological and molecular properties and signalling pathways involved. This review focuses on what we know so far about the EML4-ALK fusion variants and aims to highlight how future research can broaden our understanding, and hopefully lead to better, more personalised therapeutics.

## 2. Human EML Family and EML4

Echinoderm microtubule-associated protein (EMAP), expressed in sea urchins, was the first member of the EMAP-like (EML) protein family to be identified [9]. It was isolated as a major component of microtubule (MT) preparations from sea urchin eggs and also found to co-localise with tubulin during interphase and mitosis. Since then, orthologues have been identified across the animal kingdom, most of which associate with microtubules and contribute to the regulation of MT assembly during mitosis, although the mechanisms have not been fully elucidated. 

Humans express a family of six EML proteins, EML1 to EML6, and these are split into two subfamilies according to their protein domain structure [10]. EML1 to EML4 have an N-terminal coiled-coil domain, followed by a C-terminal domain containing a hydrophobic EML protein (HELP) domain and variable tryptophan-aspartic acid (WD) repeats [11]. These are separated by an unstructured basic linker region, rich in Ser and Thr residues. EML5 and EML6, on the other hand, lack an N-terminal coiled-coil domain and instead, have three repeats of the HELP-WD domain. EML4, initially named Ropp120, was identified through the isolation of microtubule-associated proteins from HeLa cells in 1979 [12]. EML4 shares 57% homology with EMAP and shows wide-spread tissue expression, which can also be as multiple isoforms [13].

Two recent structural studies have provided key insights into features of the EML protein family [14,15]. The first study examined the structure of the C-terminal domain of EML1, revealing a tandem atypical β-propeller in EML protein (TAPE) domain [14]. This is a highly-ordered structure where the WD repeats form 13 individual β-sheets resulting in two closely associated β-propellers with the HELP domain forming part of the hydrophobic core [14]. In the second study, the structure of the coiled-coil region of EML2 and EML4 revealed that these regions were required for trimeric oligomerisation, and this was named the trimerisation domain (TD) [15]. It is through the TD and the basic region that EML proteins are able to associate with microtubules, although it is not yet known whether this is through direct binding or via oligomerisation [15,16]. In addition, the TAPE domain is able to bind soluble α/β-tubulin heterodimers through its concave surface, but again, further work is required to understand the importance of this interaction [14].

## 3. EML4-ALK

ALK is a member of the insulin receptor superfamily, and was first identified through fusion with nucleophosmin (NPM) in anaplastic large-cell lymphoma [17]. Activation of oncogenic signalling pathways through ALK in cancer cells can occur through either activating point mutations, gene amplification or, of interest in this review, gene fusion events. In gene fusions, the subsequent localisation, activity, and expression of ALK is determined by the fusion partner.

The identification of EML4-ALK fusion protein came about in 2007, by Soda et al. [7], in an attempt to understand the molecular events involved in lung cancer development. Of the patients with NSCLC tested, the fusion transcript was identified in 6.7%, and this was mutually exclusive to those patients with epidermal growth factor receptor (EGFR) mutations [7,18]. It was also shown in this study that the EML4-ALK fusion protein possessed transforming properties both in vitro and in vivo [7,19].

**Figure 1:**
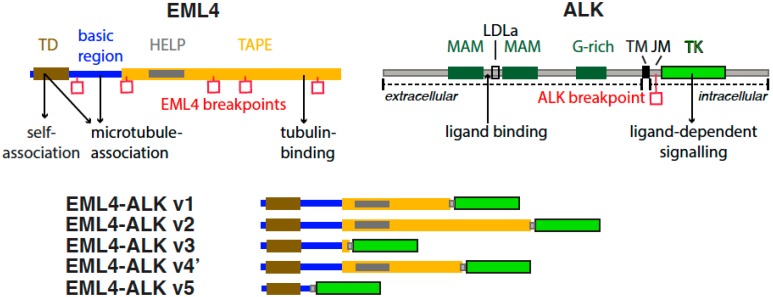
Protein domain structures and functional motifs of echinoderm microtubule associated protein-like 4 (EML4), anaplastic lymphoma kinase (ALK), and EML4-ALK fusions. EML4 domains and motifs shown are: trimerisation domain (TD); hydrophobic motif in EML proteins (HELP); tandem atypical propeller domain (TAPE). ALK domains and motifs shown are: Meprin, A5 protein, and protein tyrosine phosphatase Mu domain (MAM); low-density lipoprotein receptor class A (LDLa); glycine-rich region (G-rich); transmembrane helix (TM); juxtamembrane domain (JM); tyrosine kinase domain (TK). Key interactions and functions are marked with arrows. Breakpoints in EML4 and ALK that generate EML4-ALK fusions are marked with red squares. Longer variants of the EML4-ALK fusion protein include an incomplete TAPE domain (e.g., v1, v2, v4’), whereas shorter variants include a few residues from the TAPE domain (v3) or no TAPE domain at all (v5).

The EML4-ALK fusion protein is expressed in 2–9% of lung adenocarcinomas, and has also been identified in breast and colorectal cancers [7,20,21,22]. The translocation occurs through a paracentric inversion between EML4 and ALK genes located in the short arm of chromosome 2. To date, at least 15 EML4-ALK variants have been identified with some variants being expressed as multiple isoforms [7,21,23,24,25,26]. All variants contain the entire intracellular kinase domain of ALK, encoded by exons 20 through to 29, but differ in the point of fusion with the EML4 gene (Figure 1). More specifically, all variants contain the TD of EML4 required for the constitutive activation of ALK through oligomerisation and autophosphorylation [15]. Variants then differ in the amount of linker region and TAPE domain present, with some variants expressing no TAPE domain, and others having different lengths of it. Consequently, these differences result in the EML4-ALK variants exhibiting different biological and molecular properties, and a better understanding of these differences could help to improve patient treatment in the future (Table 1).

## 4. EML4-ALK Variants

### 4.1. Frequency

Variants 1, 2, and 3a/b are the most common EML4-ALK variants expressed in NSCLC, accounting for around 90% of all variants [29]. On average, variants 1, 2, and 3a/b are expressed in 33, 10, and 29% of positive EML4-ALK cells, respectively, with a recent report finding expression of variant 3a/b being as high as 44.4% [27,30,31].

### 4.2. Structure, Stability and Sensitivity

EML4-ALK variants 3a/b and 5a/b are the only variants that completely lack the C-terminal structured TAPE domain of EML4. Variants 5a/b are the shortest variants and also lack the basic region of EML4; however, they still exhibit transforming capabilities due to the presence of the TD required for the activation of ALK [24]. The TAPE domain is present in the remaining variants but in varying proportions. Disruption of this domain in 70% of EML4-ALK fusions contributes to key biological differences between those that lack the TAPE domain and those that have a partial one.

Comparison of endogenous EML4-ALK variant properties in cells have been carried out in a limited panel of patient-derived cell lines, and to date, cell lines available express either variant 1 or 3 only [32]. Variant 1 expressed in H3122, STE-1, and DFC1032 cells and variant 3a/b expressed in H2228 cells were generated from adenocarcinomas, and have proved useful tools for basic research, although conclusions drawn about the variants should be carefully considered due to differences in the genetic backgrounds of the cells. Study of the remaining variants in vitro are through overexpression in model cell lines.

In vitro studies show that the expression of a partial TAPE domain, such as in EML4-ALK variants 1 and 2, results in an unstable protein, whereas variants lacking a TAPE domain, such as variant 3a/b, are stable [28]. In addition, fusion variants that have a partial TAPE domain also show greater sensitivity towards inhibitors against ALK and HSP90 [14,15,28]. This is thought to be the case due to the presence of an incomplete TAPE domain making the protein unstable, and therefore requiring additional chaperones for stability [14].

### 4.3. Localisation and Microtubule Binding

EML proteins are able to bind to microtubules through the TD and basic region, and to soluble tubulin via the TAPE domain [14,15]. Fusion of EML4 to the ALK kinase domain causes mis-localisation of ALK from the membrane to regions within the cell, although it is not yet clear if this is responsible and required for ALK signalling. To date, there has been limited data collected on the localisation of endogenous EML4-ALK variants due to limited patient-derived cell lines available. Localisation studies of endogenous EML4-ALK variant 1 and 3a/b in H3122 and H2228 cells, respectively, have shown that variant 3 localises to microtubules in a way similar to EML4. Variant 1 on the other hand, shows cytoplasmic localisation [14,15].

Overexpression of EML4-ALK variants 1, 2, and 5a, in both HeLa and NIH3T3 cells, revealed cytoplasmic localisation, matching that seen for endogenous variant 1 [15]. Overexpression of variant 3, however, showed equal distribution across the cytoplasm and nucleus in NIH3T3 cells, but localisation to microtubules in HeLa cells [15,28].

Interestingly, all EML4-ALK variants have the TD responsible for EML4 localisation to microtubules, yet it is only in variant 3 that this pattern is observed [15]. It would be interesting to see why this is the case and how this affects the downstream activation of signalling pathways.

## 5. Signalling Pathways

EML4-ALK translocation is thought to lead to the activation of oncogenic signaling through multiple pathways through ALK interacting partners such as PI3K/Akt, JAK/STAT, and RAS/RAF/MEK/ERK [33].

To date, studies of EML4-ALK oncogenic signaling pathways have utilized cell lines derived from NSCLC patients or recombinant model cell lines expressing variant EML4-ALK fusions: for example, H3122 cells express EML4-ALK variant 1, whilst the H2228 cell line expresses variant 3a/b [32]. ALK inhibitors such as TAE684 and crizotinib were used in these cell lines to probe the effects on phosphorylation of downstream partners, showing that EML4-ALK signals through Akt, ERK, and possibly STAT3. Cell lines expressing different EML4-ALK variants show varying sensitivity to ALK inhibitors such as TAE684 and crizotinib. Furthermore, the stability of the fusion proteins was differently affected by ALK inhibition in a comparable pattern: variant 2 exhibited greater sensitivity than the intermediate variant 1, while variant 3a was the least affected [28].

A more detailed picture of the signaling network downstream of EML4-ALK has emerged from a study that utilized proteomics to generate a rich dataset of EML4-ALK variant 1 interactions and phosphorylation events in H3122 cells, and how they are altered by crizotinib [34]. The adaptor proteins GRB2 and SHC1 were identified as crizotinib-sensitive interaction partners of EML4-ALK variant 1 and the viability of H3122 and STE1 cells were decreased by siRNA knockdown of these two adaptor proteins. The pattern of changes in cellular tyrosine phosphorylation upon treatment of H3122 cells with crizotinib are complex: although the phosphorylation of EML4-ALK and associated proteins were reduced as expected, in addition to the other major target of crizotinib, MET, other networks of proteins showed an increase in phosphorylation. These results highlight the complex nature of signaling networks based on kinases and phosphatases. One of these upregulated networks was centered on ROCK2, and although ROCK inhibitors did not sensitize H3122 cells to crizotinib, they were sensitized to the second generation ALK inhibitor alectinib. This study illustrates the potential of network-level analysis to uncover mechanisms of drug sensitivity and to identify targets for combination therapy with ALK inhibitors that could prolong patient response.

## 6. In the Clinic

Patients expressing EML4-ALK generally have distinct clinical features including young age of onset, an absence of, or light smoking history, and are mostly adenocarcinoma in histology [32,35]. For patients with advanced disease, cytotoxic chemotherapy is often the treatment selected, but this exhibits high toxicity and poor efficacy. Due to a greater understanding of the molecular events involved in lung cancer, currently all NSCLC patients are genetically subtyped to allow for more personalised therapy, and this has proven to result in prolonged PFS.

### 6.1. ALK Inhibitors

Current therapies for NSCLC patients include tyrosine kinase inhibitors such as erlotinib in the presence of an EGFR mutation, or ALK tyrosine kinase inhibitors, crizotinib, ceritinib, and alectinib against EML4-ALK fusions [36]. Currently, there are eight second- and third-generation ALK inhibitors in clinical investigation and others are in preclinical research [37]. EML4-ALK-positive patients show around a 61–74% response rate to ALK inhibitors, such as crizotinib [6,20,38,39,40]. While this is remarkable and has had a positive impact for many patients, there is a heterogeneous response with some patients showing little or no response to treatment. In addition, it is common that initially responsive patients regress within a year post-treatment due to the acquisition of secondary mutations and the activation of alternative pathways. Therefore, current research focuses on why a proportion of patients might show no response, as well as on the mechanisms behind resistance in order to help understand why there is such variation in the duration of response between patients, with the hope that we can improve patient treatments and outcome.

Clinical studies on the response to ALK inhibitors in EML4-ALK-positive cells have largely been carried out with no discrimination between the EML4-ALK variants being expressed, and currently the majority of the studies comparing the variants have been carried out in vitro. Due to the varied response seen in EML4-ALK-positive patients to ALK inhibitors, and taking into account differences in structural stability and sensitivity between variants, researchers examined whether there was a correlation between the EML4-ALK variant being expressed and the subsequent patient response.

The first patient-based study that identified specific variants was carried out in 2010, but the patient cohort was small and included only one patient expressing variant 2, the least stable EML4-ALK variant [20]. However, this patient did show a 57% response to crizotinib, compared to the response rates below 10% seen with traditional chemotherapies [20,41].

Three retrospective studies, published in 2016 by different groups, came to different conclusions when comparing patient response to the EML4-ALK variant expressed [42,43]. Cha et al. and Lei et al. concluded that there was no significant difference in the response rate to ALK inhibitors between the ALK fusion variant [42,44]. Yoshida and colleagues, on the other hand, found that patients with variant 1 showed a better response to crizotinib than patients with other EML4-ALK variants [43].

The most recent retrospective patient study subdivided subjects into two groups: patients with variants 1/2/others and those with variants 3a/b, based on previous data on the stability of EML4-ALK variants [14,28,31]. Interestingly, patients harbouring shorter variants that lacked the TAPE domain, such as variants 3a/b or 5a, exhibited decreased response to TKI treatment when compared to variants with variable portions of the TAPE domain, such as variants 1 and 2 [31]. The two-year PFS rate for EML4-ALK variants 1/2/others was markedly greater compared to that of variants 3a/b and 5, providing key evidence that variants lacking a TAPE domain are responsible for the resistance to TKI seen in some patients. This enables us to further stratify patients and predict more precisely the treatment response and strategies that should be taken.

### 6.2. Hsp90 Inhibitors

Patients treated with crizotinib as the first-line of therapy often become resistant to treatment within a year, due to secondary mutations in the kinase domain of ALK, or in some cases, amplification of the ALK fusion gene [8]. It is known that many oncogenic kinases rely on additional chaperone proteins, such as HSP90, for stability. Encouragingly, this also seems to be the case for EML4-ALK. One of the first studies demonstrating this was carried out in 2011, where researchers generated crizotinib-resistant H3122 cells, and showed that they were sensitive to 17-AAD, an inhibitor against HSP90 [35]. However, like with ALK inhibitors, unfortunately not all patients respond to HSP90 inhibition in the clinic [45]. In vitro work by Richards et al. revealed that the structurally unstable EML4-ALK variants, such as variant 1, exhibited degradation in response to treatment with the HSP90 inhibitor ganetespib. Conversely, the more stable variants 3a/b, which lack the TAPE domain completely, were resistant [14]. This is important as it identifies a subset of EML4-ALK patients that may benefit from HSP90 inhibitors more so than others.

## 7. Discussion

Since the identification of EML4-ALK expression in NSCLC patients and the heterogeneous responses observed in terms of sensitivity and time before regression, research focus has shifted in recent years to examine how variants of EML4-ALK correlate to the responses seen. In vitro work using limited patient-derived cell lines have been useful tools in determining differences in both the stability of individual variants as well as sensitivities towards inhibitors against ALK and HSP90. Recent structural studies have helped to explain why such differences occur, with variants expressing truncated portions of the TAPE domain exhibiting greater sensitivity towards such inhibitors compared to variants completely lacking the TAPE domain. We are just beginning to understand the relevance of particular variant expression in patients through retrospective clinical studies and, interestingly, it is emerging that patients expressing variants 3a/b or 5a/b may exhibit less sensitivity to therapeutic treatments, such as crizotinib. It will be interesting to see whether these differences are more or less apparent in patients who are treated with next-generation ALK inhibitors such as alectinib or ceretinib.

It is important now to collect more patient samples to compare variant expression and subsequent response as well as to record long-term responses. In addition, the current panel of cell lines used are limited and, whilst they are generated from patients with adenocarcinomas, they will inevitably show variation in genetic background and, potentially, the signalling pathways activated. There is therefore a need to generate isogenic lung adenocarcinoma cell lines expressing EML4-ALK variants in order to compare pathways activated in cells with the same genetic background. It is also important to expand our current patient-derived cell database to include more lines that express each of the variants; these can then be used to explore new strategies to improve therapeutics for patients based on specific EML4-ALK variants.

## 8. Conclusions

EML4-ALK fusion proteins have some properties that are shared with their normal parents: they inherit microtubule binding and trimerisation from EML4, as well as kinase activity from ALK. In addition, they have properties that are unique, such as dependence on Hsp90 arising from the disruption of the TAPE domain. The properties of EML4-ALK that differ from the parental proteins merit further study, as they underpin the novel biochemical properties of the fusion protein that drives oncogenic signalling, and provide opportunities for new therapeutic strategies. Moreover, the differences between the distinct types of EML4-ALK variant proteins also bear further scrutiny as this may have consequences for how these patients respond to ALK inhibitors, and may inform strategies to optimize their outcomes.

## Figures and Tables

**Table 1:** Most common EML4-ALK variants and their properties.

EML4-ALK Variant	Gene Fusion Points	Frequency	TAPE Domain	Inhibitor Sensitivity	Localisation	References
Variant 1	E13; A20	33%	Partial TAPE	ALK- mid HSP90- high	Cytoplasm	[7,15,27,28]
Variant 2	E20; A20	10%	Partial TAPE	ALK- high HSP90- high	Cytoplasm	[7,15,27,28]
Variant 3a/b	E6a; A20	29%	No TAPE	ALK- low HSP90- low	Microtubules, cytoplasm and nucleus	[15,27,28]
Variant 4’	E14; ins11del49A20	3%	Partial TAPE	Not known	Not known	[24,27]
Variant 5a	E2; A20	2%	No TAPE	ALK- low HSP90- low	Cytoplasm	[15,24,27]
